# SPARC gene deletion protects against toxic liver injury and is associated to an enhanced proliferative capacity and reduced oxidative stress response

**DOI:** 10.18632/oncotarget.9456

**Published:** 2016-05-18

**Authors:** Estanislao Peixoto, Catalina Atorrasagasti, Mariana Malvicini, Esteban Fiore, Marcelo Rodriguez, Mariana Garcia, Paola Finocchieto, Juan J. Poderoso, Fernando Corrales, Guillermo Mazzolini

**Affiliations:** ^1^ Gene Therapy Laboratory, Instituto de Investigaciones Médicas Aplicadas-CONICET (Consejo Nacional de Investigaciones Científicas y Técnicas), Buenos Aires, Argentina; ^2^ Instituto INIGEM, UBA-CONCET, Buenos Aires, Argentina; ^3^ CIMA, University of Navarra, Pamplona, Spain

**Keywords:** SPARC, hepatocyte proliferation, reactive oxygen species, liver damage, PCNA

## Abstract

SPARC, also known as osteonectin and BM-40, is a matricellular protein with a number of biological functions. Hepatic SPARC expression is induced in response to thioacetamide, bile-duct ligation, and acute injuries such as concanavalin A and lipopolysacharide (LPS)/D-galactosamine. We have previously demonstrated that the therapeutic inhibition of SPARC or SPARC gene deletion protected mice against liver injury. We investigated the mechanisms involved in the protective effect of SPARC inhibition in mice. We performed a proteome analysis of livers from SPARC^+/+^ and SPARC^−/−^ mice chronically treated with thioacetamide. Catalase activity, carbonylation levels, oxidative stress response, and mitochondrial function were studied. Genomic analysis revealed that SPARC^−/−^ mice had an increased expression of cell proliferation genes. Proteins involved in detoxification of reactive oxygen species such as catalase, peroxirredoxine-1, and glutathione-S-transferase P1 and Mu1 were highly expressed as evidenced by proteome analysis; hepatic catalase activity was increased in SPARC^−/−^ mice. Oxidative stress response and carbonylation levels were lower in livers from SPARC^−/−^ mice. Hepatic mitochondria showed lower levels of nitrogen reactive species in the SPARC^−/−^ concanavalin A-treated mice. Mitochondrial morphology was preserved, and its complex activity reduced in SPARC^−/−^ mice. In conclusion, our data suggest that the protection associated with SPARC gene deletion may be partially due to a higher proliferative capacity of hepatocytes and an enhanced oxidative stress defense in SPARC^−/−^ mice after liver injury.

## INTRODUCTION

Acute and chronic liver damage is a growing worldwide health problem due to the increasing incidence of toxic insults such as alcohol abuse, drugs or metabolic syndrome. Hepatic toxic damage usually induces cell death signaling pathways, which may result in apoptosis and/or necrosis of hepatocytes. However, the mechanisms determining the type of hepatocyte death are not fully understood yet.

Liver damage of different causes induces secreted protein, acidic and rich in cysteine (SPARC) expression [1–5]. SPARC has the ability to induce a positive feedback with transforming growth factor beta (TGF-β)-1, stimulates collagen deposition, induces inflammation, and has anti-proliferative properties. We have previously demonstrated that downregulation of SPARC has the capacity to reduce acute liver damage after concanavalin A (ConA) administration [5], and the degree of fibrosis in tioacetamide (TAA) and bile duct ligation models [5, 4]. The mechanistic role of SPARC in liver pathologies has not been addressed, although our previous works demonstrated that SPARC^−/−^ mice treated with ConA showed reduction in T CD4^+^ lymphocytes infiltration, tumor necrosis factor alpha (TNFα) production, and apoptosis. SPARC knockdown of human microvascular endothelial cells (HMEC-1) showed reduced ConA-induced autophagy and apoptosis [5]. In two fibrogenic models generated by TAA administration and by bile duct ligation, SPARC^−/−^ mice showed a reduction in the degree of inflammation, deposits and expression levels of collagen, and in the number of activated myofibroblasts [4, 7].

Thioacetamide is a toxic compound that produces apoptosis and necrosis of hepatocytes by the generation of reactive oxygen species (ROS). Chronic TAA administration leads to fibrosis and cirrhosis [8]. Metabolization of TAA leads to the formation of reactive metabolites such as thioacetamide-S-oxide and ROS [9–11]. ROS production is followed by lipid peroxidation, calcium mobilization, glutathione depletion, and reduction in the SH-thiol groups [11–15]. ROS and calcium activate mechanisms related to reparation of cell damage or proliferation [11, 16], but might also suppress mitochondrial function [17].

In the present work we aimed to study the role of SPARC on liver damage and find the mechanisms involved in its protective effect. For this purpose, we performed a proteomic analysis of livers treated with TAA for 10 weeks. Microarray analysis showed that SPARC^−/−^ mice overexpressed a representative group of genes related with cell proliferation, and the proteomic analysis depicted overexpression of proteins related with detoxification of ROS. These results were confirmed in acute and chronic models of hepatic injury. The levels of proliferating cell nuclear antigen (PCNA) increased one day after TAA administration. Additionally, primary culture of hepatocytes from SPARC^−/−^ mice showed decreased levels of ROS, and mitochondria from SPARC^−/−^ mice treated with ConA were well preserved and showed an increased activity when compared with untreated controls.

## RESULTS

### SPARC^−/−^ mice showed an increased expression of PCNA after TAA damage

**Figure 1:**
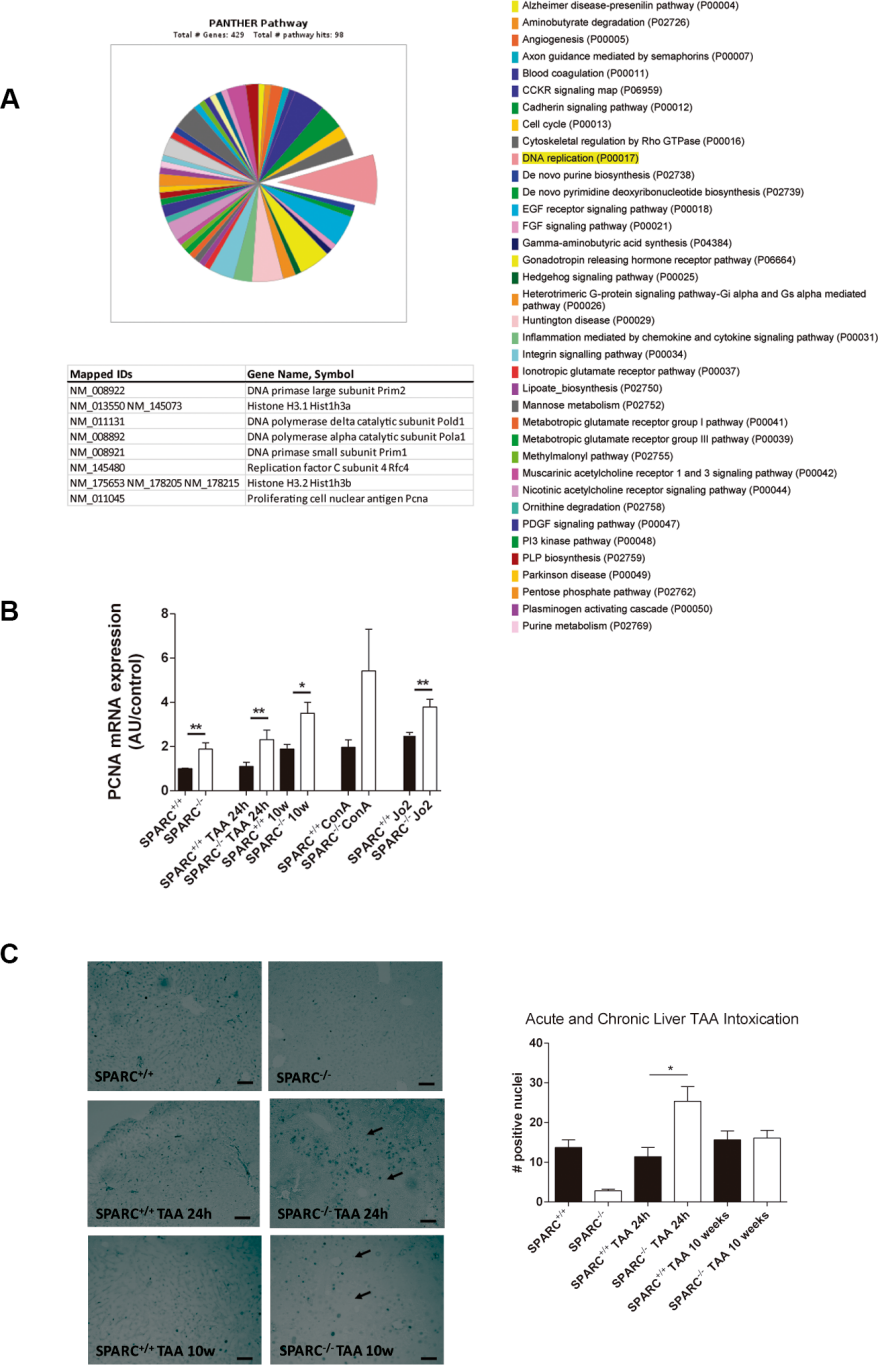
SPARC^−/−^ mice revealed increased expression of DNA replication genes. (**A**) Panther pathway chart highlighting the DNA replication GO term, and its list of genes. (**B**) PCNA transcript levels of mice injected with TAA 24 h (*n* = 3), TAA during 10 weeks (*n* = 5), ConA (*n* = 4) or anti-CD95 Jo2 (*n* = 3) measured by qPCR.*p < 0.05, **p < 0.01. Mann-Whitney test. (**C**) Immunohistochemistry for PCNA of SPARC^+/+^ and SPARC^−/−^ mice, treated or non-treated with TAA for 24 h (acute model) or 10 weeks (200×, bar 50 μm) (left panel) and its quantification (right panel); *p < 0.05. Kruskal-Wallis test, *n* = 3.

We have previously demonstrated that SPARC^−/−^ mice showed a reduced degree of liver fibrosis after 10 weeks of thioacetamide administration (Supplementary Figure 2) [4, 6]. Cell proliferation genes were highly represented on a previous microarray analysis [4]. As is showed in Figure 1, the gene ontology (GO) pathway chart revealed that DNA replication was the most affected category (Figure 1A). Therefore, we decided to assess the expression of the essential proteins for DNA replication, PCNA, by qPCR and by immunohistochemistry. As expected, SPARC^−/−^ mice treated for 10 weeks with TAA showed augmented levels of PCNA mRNA compared to SPARC^+/+^ mice. We also studied the expression of PCNA at the onset of liver injury, and observed that 24 h after TAA injection the levels of hepatic PCNA were increased in SPARC^−/−^ mice in comparison with SPARC^+/+^ mice. Importantly, same results were achieved in 2 other models of acute liver damage, ConA and the CD95 Jo2 agonistic antibody in SPARC^−/−^ mice (Figure 1B). The presence of PCNA was confirmed by immunohistochemistry. SPARC^−/−^ mice showed higher number of positive nuclei at 24 h after TAA treatment compared to SPARC^+/+^ mice, while untreated animals and 10 weeks TAA-treated animals did not reached statistically differences between SPARC^+/+^ and SPARC^−/−^ mice (Figure 1C).

### Proteome analysis of livers from SPARC knockout mice chronically treated with TAA revealed an increased detoxification of oxygen reactive species profile

**Figure 2:**
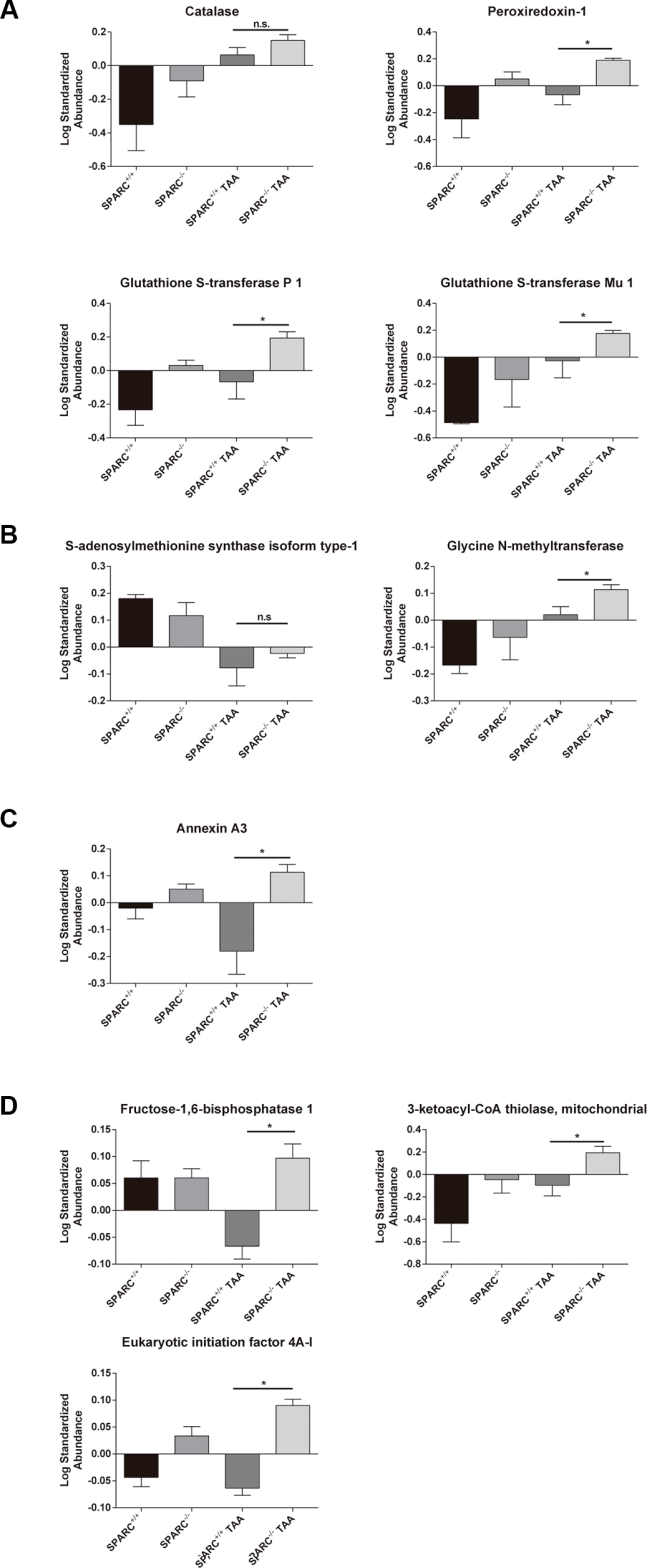
Hepatic proteome profiling revealed the induction of detoxification of oxygen reactive species and liver regeneration pathways in animals chronically exposed to TAA. (**A**) Proteins involved in detoxification of reactive oxygen species; (**B**) proteins in methyl transfer reactions; (**C**) proteins involved in regulation of cellular growth; (**D**) protein metabolism. *p < 0.05, Mann-Whitney test, *n* = 3.

A proteomic profiling of liver homogenates from SPARC^+/+^ and SPARC^−/−^ mice treated with TAA for 10 weeks was performed. As a result, we interestingly found 4 proteins related with the detoxification of reactive oxygen species (ROS) that were significantly (*p* < 0.05) upregulated in SPARC^−/−^ mice: Catalase (CAT), Peroxiredoxin-1 (PRDX1), Glutathione S-transferase P1 (GSTP1), and Glutathione S-transferase Mu1 (GSTM1, Figure 2A). Other proteins involved in the protective effect in SPARC^−/−^ mice are related with methyl transferase activity: S-adenosylmethionine synthase isoform type-1 (MAT1A) and Glycine N-methyltransferase (GNMT, Figure 2B), with regulation of cellular growth: Annexin A3 (ANXA3, Figure 2C), and metabolism: fructose-1,6-bisphosphatase 1 (FBP1), 3-ketoacyl-CoA thiolase (ACAA2) and eukaryotic initiation factor 4A-I (EIF4A1, Figure 2D).

### Low levels of endogenous ROS production in SPARC^−/−^ mice after TAA injection

**Figure 3:**
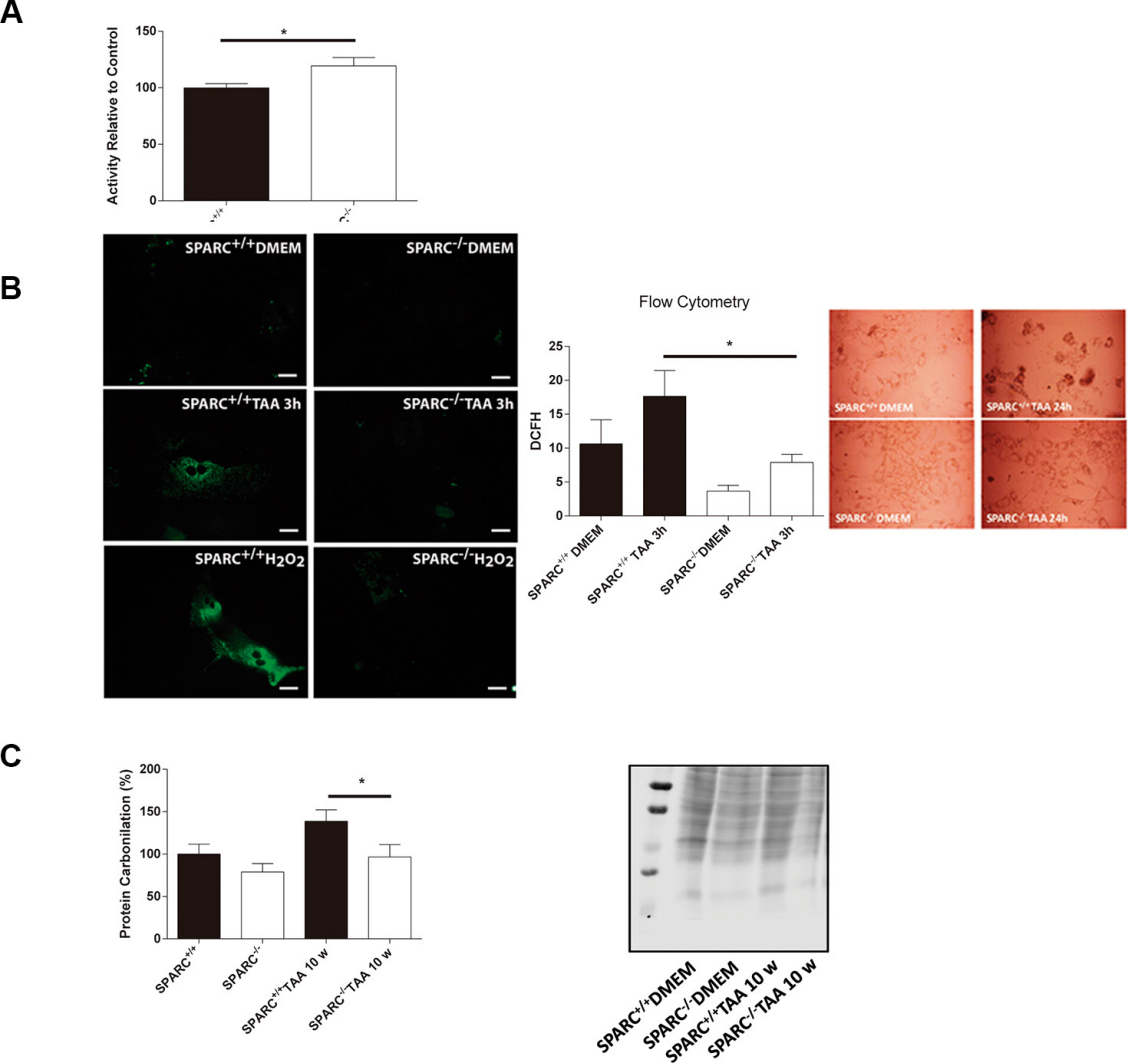
Endogenous reactive oxygen species production in response to TAA treatment. (**A**) Catalase activity of livers from SPARC^−/−^ (*n* = 6) and SPARC^+/+^ (*n* = 7) mice relative to controls. *p < 0.05, Mann-Whitney test. (**B**) Representative microphotographs of hepatocytes treated with TAA or H_2_O_2_ for 3 hours (400×, bar 25 μm) (left panel); 2′,7′-dichlorofluorescin diacetate assay for flow cytometric measurement of reactive oxygen species of hepatocytes treated with TAA for 3 hours. *p < 0.05. Mann-Whitney test, *n* = 4; photomicrographs of primary culture hepatocytes TAA-treated from SPARC^+/+^ and SPARC^−/−^ mice (400×, inset); (**C**) Carbonylation levels of liver total proteins from SPARC^+/+^ and SPARC^−/−^ mice TAA-treated for 10 weeks *n* = 4.

Uncontrolled ROS production and failure in the mechanisms of detoxification have a deleterious effect on liver function. As the proteome output shows genes implicated in ROS detoxification, we aimed to evaluate the importance of the ROS detoxification capacity of the SPARC^−/−^ liver by analyzing catalase activity. We observed that hepatic catalase activity was higher in SPARC^−/−^ mice compared with SPARC^+/+^ mice (Figure 3A). In addition, we measured the general ROS status of hepatocytes in primary culture with the cell-permeate 2′,7′-dichlorofluorescin diacetate (DCFH-DA) that is converted to a highly fluorescent compound when it reacts with reactive oxygen intermediates. As a result, primary cultured hepatocytes from SPARC^−/−^ mice showed a lower fluorescence signal in comparison with SPARC^+/+^ mice at 3 h of TAA treatment, as assessed with direct fluorescence (Figure 3B, left panel) and flow cytometry (Figure 3B, right panel). Microphotographs of hepatocytes from SPARC^−/−^ mice revealed a more preserved cell morphology (Figure 3B, Inset) when incubated with TAA for 24 h, and reduced transaminase levels than SPARC^+/+^ mice (not shown). On the other hand, as protein carbonyl groups are biomarkers of oxidative stress, we then assessed the protein carbonylation levels in liver homogenates. Importantly, SPARC^−/−^ mice presented lower levels of protein carbonylation when compared with SPARC^+/+^ mice at 10 weeks of TAA treatment (Figure 3C).

### Lack of SPARC protects mitochondrial function

**Figure 4:**
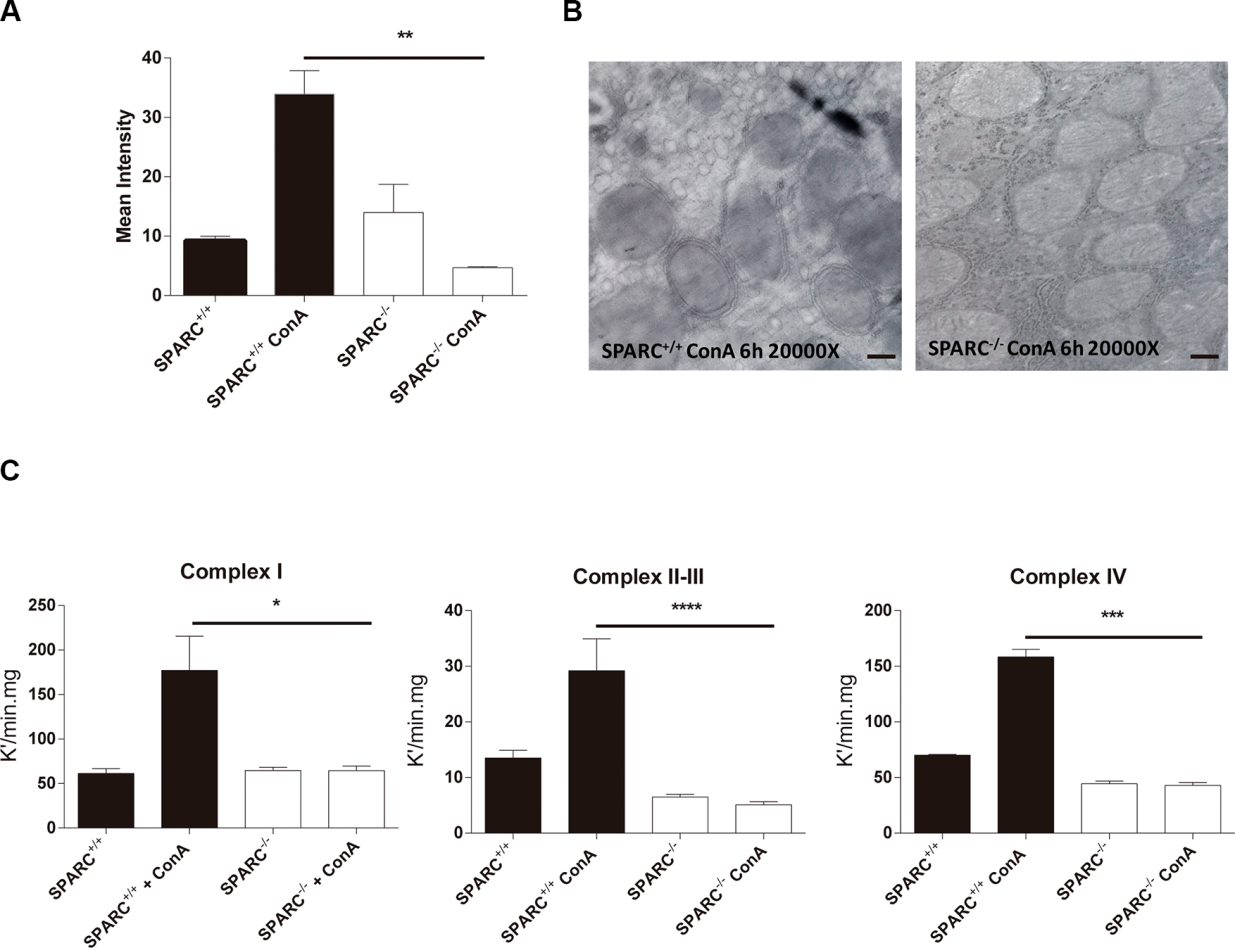
Absence of SPARC is associated with protection of mitochondrial function. (**A**) Measurement of nitric oxide levels of hepatic mitochondrias from ConA treated mice by mitotracker and DAF citometry, **p < 0.01, Kruskal-Wallis, *n* = 3; (**B**) Representative electron micrographs of hepatic mitochondrias form SPARC^+/+^ and SPARC^−/−^ mice treated ConA (20000×, bar 0.4 μm); (**C**) Mitochondrial complex I (*n* = 4), II–III (*n* = 5), IV (*n* = 5) activity. *p < 0.05, ***p < 0.001, ****p < 0.0001, Kruskal-Wallis.

Since the differences in the levels of reactive species occur also at the beginning of damage we decided to analyze the mitochondrial functionality in an acute liver injury model. Nitric oxide (NO) levels in hepatic mitochondria from ConA-treated mice were significantly higher in SPARC^+/+^ mice when compared with SPARC^−/−^ mice (Figure 4A). Interestingly, electron microscopy photographs showed that hepatic mitochondria from SPARC^+/+^ mice were wrapped in extramembranes, suggesting the presence of autophagy events (Figure 4B). Then, and considering that mitochondrial activity is related to ROS generation, we assessed mitochondrial complexes activity in hepatic tissue. As a result, we observed that the activity of complexes I, II, III, and IV were significantly increased in SPARC^+/+^ mice upon ConA exposure in comparison with knockout mice (Figure 4C).

## DISCUSSION

Our first investigations using mice deficient in SPARC showed reduced liver injury and fibrosis by thioacetamide and bile duct ligation [4]. Furthermore, SPARC^−/−^ mice were protected from acute liver failure induced by ConA and the agonistic anti-CD95 antibody Jo2 [5]. In this work, we further explored the mechanistic role of SPARC in acute and chronic liver pathologies. We have shown that SPARC^−/−^ livers exhibits decreased oxidative stress response, and higher proliferative response, which may explain at least in part the hepatic protection from injury.

Hepatocytes are normally in a quiescent G_0_ phase, and cell proliferation is a critical step to overcome liver damage due to a number of causes such as toxic exposure, partial hepatectomy, or virus infection to quickly restore its mass and function [18–20]. In line with this, our previous array analysis revealed that a number of genes related with proliferation and cell cycle were significantly over-expressed in SPARC^−/−^ mice in comparison with SPARC^+/+^. The list includes *Pcna*, an auxiliary protein of DNA polymerase delta, indicative of increased G_1_/S transition after injury [21]. Therefore, the observation that in the absence of SPARC a number of genes related with cell division are highly expressed in the liver, not only in the background but also after injury, might partially explain why its absence has a protective effect. These results are in agreement with the well known antiproliferative role of SPARC, which arrest cells at G_0_ in different cell types and pathologies including cancer cells [22–25]. The mechanisms by which SPARC inhibits proliferation were related with alteration of growth factors signaling events by diverse mechanisms including interaction with platelet derived growth factor (PDGF) receptors [26], cyclin E-Cyclin dependent kinase (CDK2) inactivation, cyclin A down-regulation, and maintenance of retinoblastoma activation [27]. Remarkably, the antiproliferative effects of SPARC were associated with indirect effects on IGF signals as observed in the kidney [28]. Altogether, our results suggest that SPARC deletion favors a more potent proliferative response of hepatocytes to toxic injury. In line with this, it has been reported that during liver remodeling phase of liver regeneration SPARC was downregulated [29]. These observations contribute to elucidate the enhanced hepatocyte proliferation observed in SPARC^−/−^ mice after injury. However, we observed that SPARC downregulation did not modify HCC growth *in vitro* [30]. Moreover, we demonstrated that down-regulation of SPARC on HepG2 cells has no effect on tumor growth *in vivo* [30].

It has been previously demonstrated that the pro-oxidative effect of TAA is the result of the generation of ROS [9–11], and that the scavenging of ROS by antioxidants prevents hepatitis after ConA treatment [31]. Our proteomic analysis showed an output of up-regulated proteins related with ROS detoxification such as catalase, peroxiredoxin-1, and Glutathion S-transferase Mu1 in the SPARC^−/−^ mice. Importantly, catalase activity was decreased *in vivo* in mice after CCl_4_ administration, and *in vitro* over-expression of catalase in hepatic stellate cells inhibited its activation state [32]; catalase has also the ability to convert hydrogen peroxide to water and oxygen decreasing ROS levels. Other hepatic protein that was identified in the proteomic study is peroxiredoxin-1, which plays a critical role in maintaining cellular redox homeostasis [33]. In agreement with these observations, glutathion S-transferase Mu1, a protein responsible for detoxification of toxins and products of oxidative stress, was also increased in SPARC^−/−^ mice in comparison with wild type mice, and probably has a synergic effect with S-adenosyl methionine to detoxify the liver of mice chronically intoxicated with TAA [34, 35]. Moreover, S-adenosylmethionine synthase isoform type-1, an important source of methyl groups for diverse biological methylations, is also upregulated in SPARC^−/−^ mice.

The absence of methionine adenosyltransferases (MATs) in mice predispose to liver injury [36]. S-adenosylmethionine is on the pathway of generation of homocysteine needed for glutathione production [34, 35]. On the same biochemical pathway glycine N-methyltransferase catalyzes the methylation of glycine using S-adenosylmethionine to form sarcosine and S-adenosylhomocysteine that is importantly upregulated in SPARC^−/−^ mice. It was previously observed that glycine N-methyltrasferase^−/−^ mice developed chronic hepatitis [36]. Therefore, the repertoire of over-expressed proteins observed in SPARC^−/−^ mice is in consonance with a reduced induction of ROS after injury.

Other remarkable proteins detected in the proteomic analysis and upregulated in SPARC^−/−^ mice in comparison with SPARC^+/+^ mice were 3-ketoacyl-CoA thiolase, an enzyme that participates in the beta-oxidation of long chain fatty acids; the eukaryotic initiation factor 4A–I, that is an ATP-dependent RNA helicase which is a subunit of the eIF4F complex and required for mRNA binding to ribosome and AnnexinA3, and was also highly expressed in SPARC null mice. It has been previously reported that Annexin A3 is expressed on regenerating livers and is stimulated by HGF [37]. However, further studies are needed to elucidate its role in our animal model. Fructose 1,6 bisphosphatase 1 that catalyzes the hydrolysis of fructose is upregulated in the SPARC^−/−^ mice. It has been observed that fructose 1,6 bisphosphate protects against D-galactosamine toxicity in isolated rat hepatocytes [38].

On the other hand, hepatic catalase activity is high in SPARC^−/−^ background mice. Considering that TAA has the capacity to induce hepatocyte necrosis, apoptosis, ROS, and reactive metabolites such as thioacetamide-S-oxide, the high catalase activity observed in SPARC null mice might explain the observed protective effects against ROS induction by TAA. The antioxidant profile observed in SPARC^−/−^ mice at the proteome level was corroborated by the changes in ROS levels in dichlorofluoresceine assay, and in carbonylation studies.

Superoxide dismutase, NADH and glutathione, and cytosolic catalase normally detoxify mitochondrial ROS. Disruption of ROS production might favors its accumulation in the mitochondria with the result of increased cellular oxidative stress. ROS has the ability to cause a variety of cellular responses from transient to permanent growth arrest, apoptosis or necrosis depending on its levels. Our results show an increased activity of the complex I, II, II and IV on the SPARC^+/+^ mice. These observations may explain the increase of ROS and mitochondria injury that was detected in electronic microphotographs of SPARC^+/+^ mice. This is consistent with the results of Socha *et al.* that reported a decreased production of reactive oxygen species in kidneys from SPARC^−/−^ mice [39]. These results were in line with the observation that SPARC may be required for hydrogen peroxide (H_2_O_2_) production in fibroblasts treated with TGF-β [40].

In summary, the present work provides evidence of an essential role for SPARC in hepatocyte proliferation, ROS induction, and liver regeneration after damage. Furthermore, this study also suggests that SPARC inhibition could be an important therapeutic target for hepatic protection in acute and chronic liver injury.

## MATERIALS AND METHODS

### Animals and experimental design

Male C57BL/6x129SvJ (The Jackson Laboratory, Bar Harbor, Maine, USA) SPARC^+/+^ and SPARC^−/−^ mice were used. Animals were administered intraperitoneally (i.p.) with 200 mg/kg of thioacetamide (Sigma, St Louis, MO) 3 times a week. Animals were sacrificed at 10 weeks after TAA application onset, and blood and liver samples were obtained. For acute liver models mice were given a single i.p. injection of TAA at 100 mg/kg or an i.v. injection of Con A (Sigma, St Louis, MO, USA) at 15 μg/g. Animals were sacrificed 24 h after TAA or Con A administration and samples were obtained (Supplementary Figure 1). Other group of animals received a sub-lethal dose (0.25 μg/g) of the agonistic anti-CD95 antibody Jo2 and killed at 24 h. All experimental procedures were performed according to the “Guide for the Care and Use of Laboratory Animals” published by the U.S. National Research Council (National Academy Press, Washington, D.C. 1996) and approved by the Animal Ethics Committee, Austral University. For *in vitro* studies TAA was used at a 100 mM concentration.

### Functional and pathway analysis

Functional enrichment analysis of Gene Ontology (GO) categories was carried out using standard hypergeometric test [41]. The biological knowledge extraction was complemented through the use of Ingenuity Pathway Analysis (Ingenuity Systems, http://www.ingenuity.com), which database includes manually curated and fully traceable data derived from literature sources.

### Reverse transcription-polymerase chain reaction (RT-PCR)

Liver tissue was homogenized and total RNA was extracted and processed as previously described [5]. For qPCR of mouse samples the mRNA levels were quantified using the following primers: PCNA (Supplementary Materials & methods). Results were depicted as arbitrary units (AU) related to control.

### Immunohistochemistry studies

Livers were immersed in 10% phosphate-buffered formalin. Fixed tissue was embedded in paraffin, sectioned (5 μm) and used for immunohistochemical analysis of PCNA expression as described in Supplementary Information.

### Two-dimensional difference gel electrophoresis (2DDIGE) and imaging

All proteomic determinations were done at the Proteomics Core Facility of the Center for Applied Medical Research (CIMA), an affiliate of ProteoRed, the Spanish National Institute of Proteomic Facilities. Protein extracts were obtained from animals treated with TAA for 10 weeks (*n* = 4) or without treatment (*n* = 4). Different samples were solubilized in 2DDIGE buffer: 7 M urea, 2 M thiourea, 4% CHAPS, and 30 mM Tris, buffered to pH 8. Protein concentration was determined using Bradford’s assay (Bio-Rad, Hercules, CA). 2DDIGE was performed as described in Supplementary Materials & methods [42, 43].

### LC-ESI-MS/MS analysis

LC-ESI-MS/MS analysis was performed as described in Supplementary Materials & methods [42, 43].

### Catalase activity

Catalase activity was measured as described by Beers *et al.* [44]. Briefly livers were homogenized using sonication and the detection of peroxide decomposition by catalase was monitored using spectrometry at 240 nm.

### Liver cell isolation and oxidative stress assessment

Hepatocytes were isolated by collagenase perfusion [45]. Hepatocytes were growth in phenol red-free Dulbecco’s modified Eagle’s medium (DMEM) plus 10% fetal bovin serum. Cells were treated with 100 mM TAA for 3 or 24 h. Oxidative stress was detected by flow cytometry after incubating hepatocytes in DMEM with using 2′,7′-dichlorofluorescin diacetate (DCFH-DA) 5 M for 30 min at 37°C with 5% CO2 [46].

### Protein carbonylation

Protein carbonylation analysis was performed as described in Supplementary Materials & methods.

### Quantification of hepatic mitochondrial nitric oxide levels

Mitochondrias (1 mg of protein per ml) were incubated in PBS for 30 min at 37°C with 5% CO_2_, 10 M 4-Amino-5-methylamino-2′,7′-difluorofluorescein (DAF-FM), and 0.5 M MitoTracker. Fluorescence was measured with an Ortho-Cytoron Absolute Cytometer (Johnson and Johnson) [46, 47].

### Electron microscopy

Con A-treated animals were sacrificed after 6 h of ConA administration and hepatic tissue were obtained as described previously [48]. Ultrathin sections (50 nm) were made and observed under a Hitachi H-7000 electron microscope (Hitachi, Tokyo, Japan).

### Mitochondrial O_2_ utilization and electron transfer activity

Oxygen (O_2_) uptake was measured polarographically with a Clark-type electrode [49]. To assess nitric oxide (NO) effects, mitochondria were incubated with 0.3 mM L-arginine (L-Arg) alone or plus 3 mM L-NMMA (a nitric oxide synthase inhibitor) for 5 min at 37°C [49]. State 4 O_2_ uptakes was determined with 6 mM malate-glutamate as substrate of complex I and state 3 active respiration by the addition of 0.2 mM adenosine diphosphate (ADP). Complex I activity (NADH: ubiquinone reductase) was measured by the rotenone-sensitive reduction of 50 μM 2,3-dimethoxy-6-methyl-1,4-benzoquinone with 1 mM KCN and 200 μM NADH as electron donor at 340 nm with a Hitachi U3000 spectrophotometer at 30°C. Activity of complexes II–III was determined by cytochrome c reduction at 550 nm. cytochrome oxidase activity (Complex IV) was determined by monitoring cytochrome c oxidation at 550 nm (є_550_, 21 mM^−1^.cm^−1^); the reaction rate was measured as the pseudo-first order reaction constant (*k*’) and expressed as nmols/min.mg protein (*K*’/min.mg of protein for complex IV) [50, 51].

### Statistical analysis

Data are expressed as mean ± SEM when appropriate. Statistical analysis was performed using Student’s *t* test or Mann-Whitney, according to value data distribution. Differences were considered to be significant when *p* < 0.05. The results shown are mean values of three independent experiments.

## SUPPLEMENTARY MATERIALS FIGURE AND TABLES


