# Supplementary material for: SPARC gene deletion protects against toxic liver injury and is associated to an enhanced proliferative capacity and reduced oxidative stress response
Source: Oncotarget. 2016 May 18;10(41):4169–79. doi: 10.18632/oncotarget.9456 (PMC6609249; doi:10.18632/oncotarget.9456)
Supplement: Supplementary file 1 [file oncotarget-10-4169-s001.pdf]

# SPARC gene deletion protects against toxic liver injury and is associated to an enhanced proliferative capacity and reduced oxidative stress response

## Supplementary Materials

## SUPPLEMENTARY METHODS

### Microarray data analysis

Both background correction and normalization were done using RMA (Robust Multichip Average) algorithm [1]. Then, a filtering process was performed to eliminate low expression probe sets. Applying the criterion of an expression value greater than 64 in 2 samples for each experimental condition, probe sets were selected for statistical analysis. R and Bioconductor were used for preprocessing and statistical analysis. LIMMA (Linear Models for Microarray Data) [2, 3] was used to find out the probe sets that showed significant differential expression between experimental conditions. Genes were selected as significant using criteria of *p*-value < 0.01.

### Reverse transcription-polymerase chain reaction (RT-PCR)

PCNA sense (5′-AAAGATGCCGTCGGGTG AAT-3′) antisense (5′-TTCCCATTGCCAAGCTCTCC-3′), were quantified by SYBR® Green (Invitrogen),. All PCR amplifications were carried out using a cycle of 95°C for 10 min and 40 cycles under the following parameters: 95°C for 30 sec, corresponding melting temperature for 30 sec, 72°C for 1 min. At the end of the PCR reaction, the temperature was increased from 60°C to 95°C at a rate of 2°C/min, and the fluorescence was measured every 15 sec to construct the melting curve. Values were normalized to levels of glyceraldehyde-3-phosphate dehydrogenase (GAPDH; used as housekeeping) transcript (forward 5′-CATCTCTGCCCCCTCTGCTG-3′; reverse 5′-GCCTGCTTCACCACCTTCTTG-3′). Data were processed by the ΔΔCt method. The relative amount of the PCR product amplified from untreated cells was set as 1. A non-template control (NTC) was run in every assay, and all determinations were performed in triplicate in two or three separated experiments.

### Immunohistochemistry studies

For chromogenic immunohistochemical analysis and quantification of PCNA expression, sections were deparaffinized, rehydrated and subject to antigen retrieval with buffer citrate (pH = 6). Endogenous peroxidase was blocked with 0.5% $H_2O_2$ in methanol. Tissue was then incubated with the rabbit anti-mouse PCNA polyclonal antibody (1:100; Santa Cruz Biotechnology, TX). After extensive washing, slides were incubated with peroxidase-linked biotinylated goat anti-mouse secondary antibodies for 1 h, washed and further incubated with avidin-peroxidase complex complex. They were then washed twice with PBS and twice with 0.1 M acetate buffer before incubation with a solution of 3.3-diaminobenzidine (DAB; Sigma), ammonium nickel sulfate and $H_2O_2$ until signal was developed. For controls slides, primary antibody incubation was omitted in control slides. Quantitative analysis of PCNA immunostained area was performed and results were expressed as number of positive nuclei.

### Two-dimensional difference gel electrophoresis (2DDIGE) and imaging

50 µg of protein was taken, labeled with 400 pmol of CyDye DIGE Fluor minimal dyes (GE Healthcare, Madrid, Spain), and incubated on ice in the dark for 30 min according to the manufacturer's instructions. Cy3 and Cy5 were used with samples; Cy2 was used with an internal control mixture composed of equal amounts of protein from all samples. Paired samples were reverse-labeled in order to prevent potential dye labeling bias. The reaction was stopped by addition of 1 µL of 10 mM lysine and incubated on ice for 10 min. Samples were up-loaded onto immobilized pH gradient strips (24 cm long, pH 3–11 NL, from GE Healthcare, Madrid, Spain), and subjected to isoelectrofocusing in the IPGphor isoelectro-focusing system System (GE Healthcare, Madrid, Spain) according to the manufacturer's recommendations. Upon completion of IEF, strips were incubated for 15 min in equilibration buffer (50 mM Tris-HCl of pH 8.8, 6 M urea, 30% glycerol, 2% SDS, and a trace of bromophenol blue) containing 0.5% dithiothreitol, and subsequently for 15 min in equilibration buffer with 4.5% iodoacetamide. For the second dimension, strips were loaded onto 12.5% polyacrylamide gels and run, at 1 W/gel, for 12–14 h., until the bromophenol blue reached the bottom end of the gel. Subsequently, 2D gels were scanned using a Typhoon Trio Imager (GE Healthcare, Madrid, Spain) at 100 λ resolution with λex/λem of 488/520

nm for Cy2, 532/580 nm for Cy3, and 633/ 670 nm for Cy5. The photomultiplier tube was set to ensure that the maximum pixel intensity was between 90,000 and 99,000 pixels. Image analysis was performed with DeCyder 6.5 software (GE Healthcare, Madrid, Spain) as specified in the user's manual and briefly summarized below. The differential in-gel analysis (DIA) module was used for spot detection, spot volume quantification, and volume ratio normalization of different samples in the same gel. The Biological Variation Analysis (BVA) module was then used to match protein spots on different gels and to identify protein spots with substantial differences. Manual editing was performed in the BVA module to ensure that spots were correctly matched on different gels and to remove streaks and speckles.

Differentially expressed spots were considered for mass spectrometry (MS) analysis if the corresponding t test $p$-value was $< 0.05$. Preparative gels were run with 350 µg of protein following the procedure described above. Proteins were visualized by staining with SYPRO Ruby Protein Gel Stain (Bio-Rad, Hercules, CA), and images were acquired with a Typhoon Trio Imager using a λex/λem of 532/560 nm. The desired spots were excised manually, and gel specimens were processed with a MassPrep station (Waters, Saint-Quentin, France) as described elsewhere (Santamaria 2003, Proc Natl Acad Sci USA). In-gel tryptic digestion was performed with 12.5 ng/µL trypsin in 50 mM ammonium bicarbonate for 12 h at 37°C. The resulting peptides were extracted with 5% formic acid and 50% acetonitrile. Samples were then concentrated in a Speed-vac before MS analysis.

### LC-ESI-MS/MS analysis

Microcapillary reverse-phase LC was performed with a CapLC (Waters, Saint-Quentin, France) capillary system. Reversed phase separation of trypsin digests was performed with an Atlantis, C18, 3 µm, 75 µm × 10 cm Nano Ease fused silica capillary column (Waters, Saint-Quentin, France) buffered in 5% acetonitrile with 0.2% formic acid. After injection of 6 µL of sample, the column was washed for 5 min with the same buffer as above, and the peptides were eluted using a linear gradient of 5–50% acetonitrile in 30 min at a constant flow rate of 0.2 µL/min. The column was coupled online with a Q-TOF Micro (Waters, Saint-Quentin, France) using a PicoTip nanospray ionization source (Waters, Saint- Quentin, France). The heated capillary temperature was 80°C and the spray voltage was 1.8–2.2 kV. MS/MS data were collected in an automated, data-dependent mode. The three most intense ions in each survey scan were sequentially fragmented by collision-induced dissociation (CID) using an isolation width of 2.5 and a relative collision energy of 35%. Data processing was performed with MassLynx 4.0. Database searching was done with ProteinLynx Global Server 2.1 (Waters) and Phenyx 2.2 (GeneBio, Geneva, Switzerland) against Uniprot knowledgebase Release 12.3 consisting of UniprotKB/Swiss-Prot Release 54.3 and UniprotKB/TrEMBL Release 37.3. The search was enzymatically constrained for trypsin and allowed for one missed cleavage site [4].

### Protein carbonylation

Liver samples were homogenized in 7M urea, 4% CHAPS, 1 mM EDTA and 50 mM Tris, pH 7.5. Cytosolic extracts were obtained by ultracentrifugation at 100,000 × g for 1 h at 10ºC. Protein concentration was measured with the Bradford assay kit (BioRad) and 25 µg protein were mixed with an equal volume of 12% SDS and incubated at room temperature (RT) for 15 min. Protein carbonylation was measured with the OxyBlotTM Protein Oxidation Detection Kit (Millipore) following the instructions of the manufacturer. Electrophoresis and blotting were done as described elsewhere [5]. Membrane was blocked with 5% DPBST-BSA for 1 h. Primary antibody was 1/75 diluted in the same buffer and incubation was carried out for 14 h at 4ºC. After five 5 min washing steps with DPBST, the membrane was incubated with the secondary antibody (goat anti rabbit IgG HRP, Sigma Aldrich) at a dilution 1/5000 in DPBST-BSA. The immunoreactivity was visualized by enhanced chemiluminiscence (Perkin Elmer).

## REFERENCES


1. Irizarry RA, Bolstad BM, Collin F, Cope LM, Hobbs B, Speed TP. Summaries of Affymetrix GeneChip probe level data. Nucleic Acids Res. 2003; 31:e15. https://doi.org/10.1093/nar/gng015. [PubMed]
2. Smyth GK. Linear models and empirical bayes methods for assessing differential expression in microarray experiments. Stat Appl Genet Mol Biol. 2004; 3:Article3. https://doi.org/10.2202/1544-6115.1027. [PubMed]
3. Gentleman R. Bioinformatics and computational biology solutions using R and Bioconductor. Statistics for biology and health. (New York: Springer Science + Business Media), pp. 1 online resource (xix, 473 p.). (2005).
4. Folio C, Mora MI, Zalacain M, Corrales FJ, Segura V, Sierrasesumaga L, Toledo G, San-Julian M and Patino-Garcia A. Proteomic analysis of chemonaive pediatric osteosarcomas and corresponding normal bone reveals multiple altered molecular targets. J Proteome Res. 2009; 8:3882–3888. https://doi.org/10.1021/pr900113w. [PubMed]
5. Sanchez-Quiles V, Mora MI, Segura V, Greco A, Epstein AL, Foschini MG, Dayon L, Sanchez JC, Prieto J, Corrales FJ, Santamaria E. HSV-1 Cgal+ infection promotes quaking RNA binding protein production and induces nuclear-cytoplasmic shuttling of quaking I-5 isoform in human hepatoma cells. Mol Cell Proteomics. 2011; 10:M111 009126. https://doi.org/10.1074/mcp.M111.009126. [PubMed]

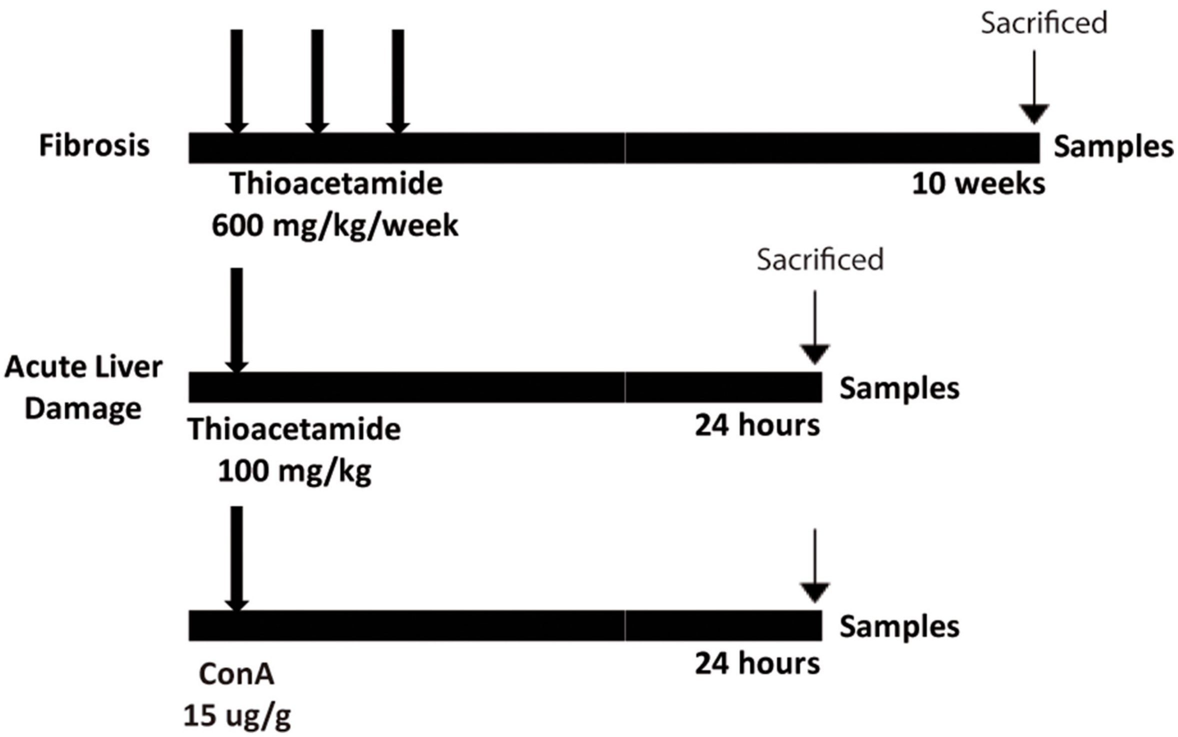


**Supplementary Figure 1: Applied models for liver damage.** Thioacetamide was administrated three times per week for 10 weeks at a 600 mg/kg dose for the generation of fibrosis. For the acute liver damage model thioacetamide was administrated i.p. on one dose of 100 mg/kg 24 hours before sacrifice or an i.v. injection of Con A at 15 µg/g 24 hours before sacrifice.

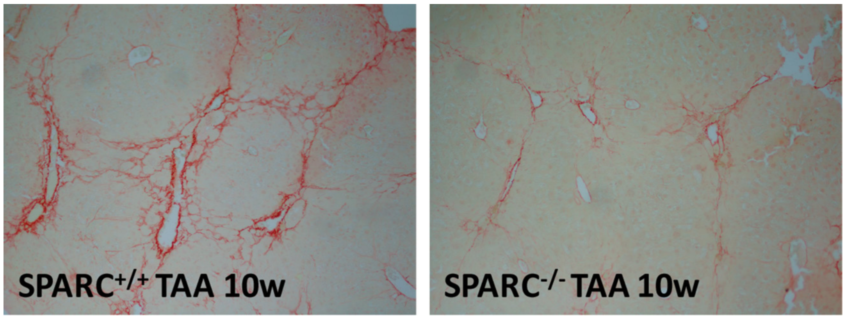


**Supplementary Figure 2: Reduced liver fibrosis in SPARC deficient mice.** Sirius Red representative microphotographs of livers from $SPARC^{+/+}$ and $SPARC^{-/-}$ mice TAA-treated for 10 weeks (100×).
